# Curiosity in the genes: the DNA fingerprinting story

**DOI:** 10.1186/2041-2223-4-20

**Published:** 2013-11-18

**Authors:** Mark A Jobling

**Affiliations:** 1 Department of Genetics, University of Leicester, University Road, Leicester LE1 7RH, UK

## 

It is unusual for a scientific field to be associated with a single individual, but in the case of the subject of the thematic series now being launched in *Investigative Genetics*, this is surely so; Alec Jeffreys (Figure 1) *is* DNA fingerprinting. Having invented the method, he coined the perfect name for it - how different things might have been if it had been called the tandem-repeat-based identification technique (or something similarly dull). He realized its potential and immediately applied, developed and refined it. He then followed his nose to unravel the mystery of the madly mutable minisatellites that make up DNA fingerprints, and eventually to understand the engines of genome variability that reside in recombination hotspots. 'I think I was born a scientist’, he has said [1]. He certainly seems to have been born with unquenchable enthusiasm and curiosity, and it is this quality that has led him on his extraordinary scientific journey.

**Figure 1:**
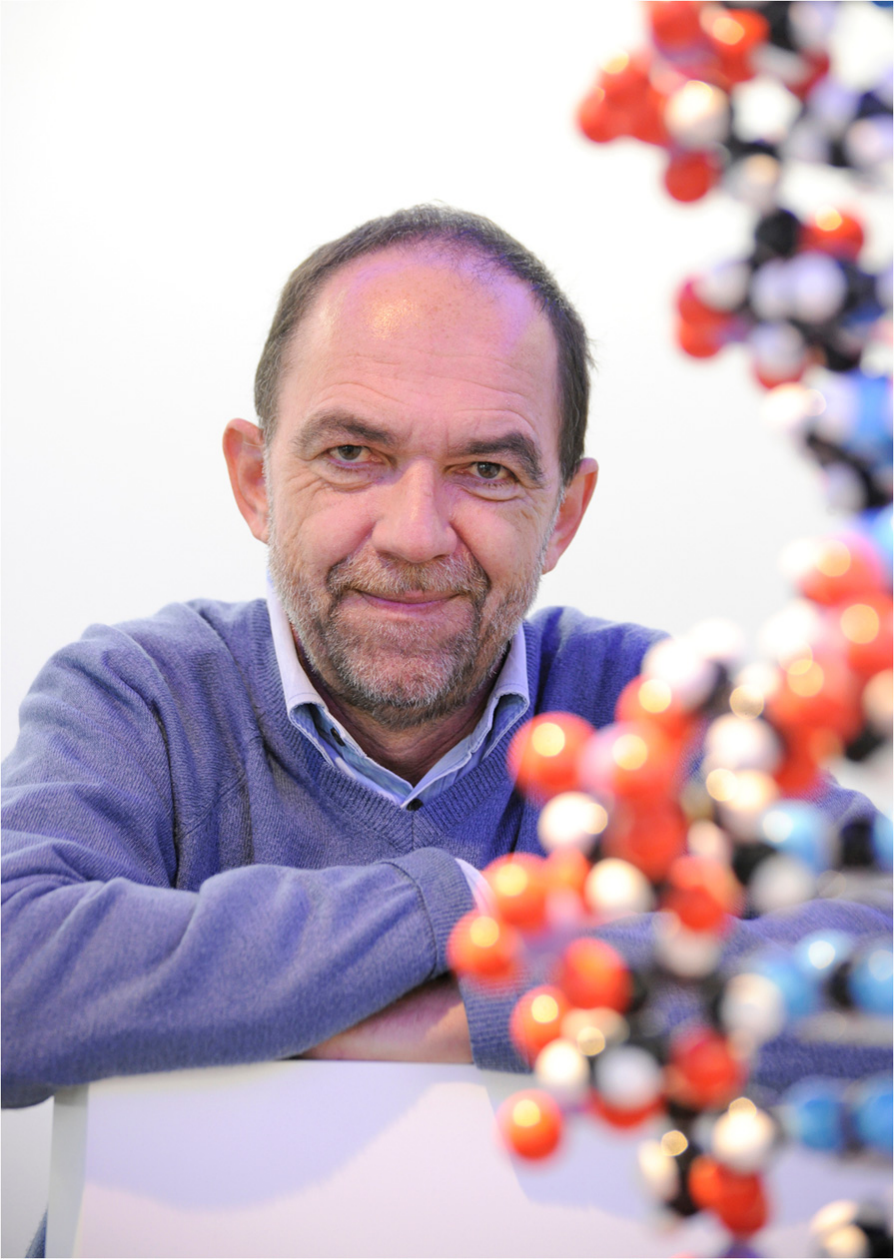
**Professor Sir Alec Jeffreys.** (Picture taken by Colin Brooks, courtesy University of Leicester) Prof Sir Alec Jeffreys has provided informed consent for the publication of his photograph.

The story of DNA fingerprinting has been told more than once, but that is because it is such a good tale (its inventor tells it very well himself [2]), and it deserves a brief retelling here. Having noticed the sequence similarity between core elements of tandem repeats in the myoglobin gene and a few other known minisatellites, Alec made a pure repeat probe, and radiolabelled and hybridized it to Southern blots of restriction-digested DNA. The probe cross-reacted with a set of hypervariable minisatellites, and on the morning of Monday 11 September 1984 the first fuzzy DNA fingerprint emerged from the developing tank. In this 'eureka moment’ , Alec could immediately see the diversity, and the pattern of inheritance in DNA from a human pedigree. In his seminal paper [3] he foresaw roles for the method in linkage analysis, in testing tumour clonality, twin zygosity and paternity, in forensic typing, and in dissecting fundamental aspects of mutation and recombination processes. In one remarkably productive year these ideas were developed in four further papers [4-7], three of them in *Nature*.

The first DNA fingerprinting application was in parentage testing [6]; normally it is the father who is in doubt, but this unusual and challenging case was a maternity test, with paternal DNA unavailable. British nurse Christiana Sarbah’s 13-year-old son Andrew was denied re-entry to the United Kingdom after a visit to Ghana, the immigration authorities suspecting that he was not her child. Given three undisputed children for comparison, it was possible to reconstruct the absent father’s DNA fingerprint, and to strongly support the claimed maternity over alternative relationships such as aunt-nephew - something that was not achieved with traditional protein polymorphisms such as blood groups. In an immigration tribunal, the UK Home Office accepted the DNA evidence, and allowed Andrew to stay with his mother and siblings. It also stated that it would not contest future immigration disputes where similar evidence was available, which effectively broke a log-jam of such cases, but created an avalanche of casework for the Jeffreys lab before the methods were commercialized [8].

Hot on the heels of this came the first application of DNA fingerprinting in forensic identification, in a case that beautifully exemplifies the power of DNA evidence to link crime-scenes, to exclude suspects, and to support convictions. Work with Peter Gill and Dave Werrett had shown that DNA fingerprints could be obtained from old samples, and importantly that the method of differential lysis could yield male-specific information from mixed rape-case samples [4]. When Leicestershire Police suggested DNA fingerprinting be applied in a local murder investigation it seemed straightforward - two 15-year-old girls had been raped and strangled about 3 years apart with the same modus operandi, and a suspect was in custody who had confessed to the second killing. As expected, DNA profiles (now based on specific cloned minisatellites, known as single-locus probes) from semen samples at both crime-scenes showed that the same man was responsible in each case. The surprise, though, was that the suspect matched neither scene - the first DNA-based exoneration. A bold police decision then triggered the first DNA-based mass screen to find the true culprit. Blood samples were taken from 5,000 local men, and following initial exclusion using protein polymorphisms, the remaining 500 were tested using the new DNA technology. None matched, but the stalemate was broken when Ian Kelly, a colleague of the perpetrator Colin Pitchfork, told friends that he had been persuaded to provide a blood sample on his workmate’s behalf. The eventual DNA profile from Pitchfork himself matched the crime-scenes, he was convicted, and he remains incarcerated today.

As well as human DNA, that first autoradiograph had included samples from various other species, and showed that the core minisatellite probes also detected hypervariable loci in non-human genomes [1]. Applications promptly followed in mice [9], cats and dogs [10], and birds [11,12]. Perhaps the last hurrah of true DNA fingerprinting came with the autoradiograph published to confirm that Dolly the sheep was indeed a clone [13].

I first encountered Alec when he gave a barn-storming talk to the 1991 International Congress of Human Genetics, in Washington DC. His subject there, described with his trademark enthusiasm, was a near-magical trick that detected and mapped the sequence variation between individual repeat units within a minisatellite, revealing a mind-boggling degree of diversity. He had embraced PCR early, showing that profiling could be done from trace amounts of DNA [14], then developing his improbable method - minisatellite-variant-repeat (MVR) PCR [15]. With a Y-chromosomal minisatellite in hand [16], I wanted to try it too, and came to Leicester in 1992, finding the same hospitable environment that Alec discovered fifteen years earlier, and, in Alec himself, a generous sponsor. MVR-PCR was perhaps the ultimate DNA fingerprint, but instead of impacting on forensic analysis, it turned out to be a key tool in understanding the complex recombination processes that drive minisatellite diversity [17]. Forensic DNA testing was moving towards short tandem repeats (STRs), and after using these markers in a collaboration with Erika Hagelberg to identify the skeletal remains of a murder victim [18], and of Josef Mengele [19], Alec’s research interests shifted away from forensics. He has, however, maintained his willingness to engage in public debate about forensic genetics and DNA databases.

Alec himself deplores the bean-counting that goes on in judging science these days, but nonetheless it is worth noting that he has well over 200 publications, an h-index of 67, and over 21,000 citations of his work, a figure that continues to grow. Since joining the University of Leicester in 1977, he has enhanced the reputation of the place enormously, as can be confirmed by a casual glance at almost any piece of University publicity. It was something of a surprise when he retired in September 2012 since his enthusiasm for experimental science and his continuing technical élan seemed likely to keep him at the bench, pipette in hand, forever. Our Vice-Chancellor, during the valedictory address, noted the irreplaceability of Alec, but said that he took some comfort from the fact that 'at least we now have Richard III’ [20]. The amusement expressed by Alec at being supplanted by the skeletal and wormy [21] remains of a long-dead king can be imagined.

DNA fingerprinting has also, of course, had a massive impact on society. Indeed, it is hard to think of another modern scientist whose work has had the societal reach of Alec’s. His list of prizes and honorary degrees is almost absurdly long (particularly when set beside those of his departmental colleagues), but it is the public recognition that is particularly telling - Midlander of the Year (1989), Honorary Freeman of the City of Leicester (1993), and Morgan Stanley Greatest Briton (2007). Another honour that many UK celebrities quietly crave is an appearance on BBC Radio 4′s long-running programme *Desert Island Discs.* Participants must choose eight records they would take to a desert island and use them to punctuate an interview about their lives; it is said that many a deceased public figure is found to have their disc list stored safely away in preparation for the call. Alec was called [22], and his appearance shook middle England out of its slumber by including *Feel the Beat*, by Trance DJ 'Darude’ , among his chosen records.

As well as a spectacular researcher, Alec has been a highly valued colleague in Leicester - a modest and truly collegiate person who has been a popular teacher of undergraduates, who has shown an almost superhuman ability to unfailingly ask a pertinent question at the end of a seminar (no matter how boring or impenetrable it was), and who has been generous with his advice and support.

These days our universities, institutes and funders spend a lot of time beating us over the head with demands for our research strategies, translational research plans, and pathways to impact. As Alec has said [23], 'If someone had told me in 1980, 'Go away and figure out a way of identifying people with DNA’ , I would have sat there looking very stupid and got nowhere at all’. The story of DNA fingerprinting is a reminder that following your nose, and your scientific enthusiasm, can get you a long way.

## Competing interests

The author declares that he has no competing interests.
